# Higher levels of Bifidobacteria and tumor necrosis factor in children with drug-resistant epilepsy are associated with anti-seizure response to the ketogenic diet

**DOI:** 10.1016/j.ebiom.2022.104061

**Published:** 2022-05-19

**Authors:** Maria Dahlin, Stephanie S. Singleton, John A. David, Atin Basuchoudhary, Ronny Wickström, Raja Mazumder, Stefanie Prast-Nielsen

**Affiliations:** a Neuropediatric Department, Astrid Lindgren Children's Hospital, Karolinska Hospital, Stockholm, Sweden; b School of Medicine and Health Sciences, Department of Biochemistry and Molecular Medicine, The George Washington University, HIVE Lab, Washington, D.C. 20037, USA; c Department of Applied Mathematics, Virginia Military Institute, Lexington, VA 24450, USA; d Centre for Translational Microbiome Research (CTMR), Department of Microbiology, Tumor and Cell Biology, Karolinska Institutet, Stockholm, Sweden

**Keywords:** Gut microbiota, Inflammation, Epilepsy, Ketogenic diet, Bifidobacterium, TNF, Omics, Bioinformatics, Machine learning

## Abstract

**Background:**

Recently, studies have suggested a role for the gut microbiota in epilepsy. Gut microbial changes during ketogenic diet (KD) treatment of drug-resistant epilepsy have been described. Inflammation is associated with certain types of epilepsy and specific inflammation markers decrease during KD. The gut microbiota plays an important role in the regulation of the immune system and inflammation.

**Methods:**

28 children with drug-resistant epilepsy treated with the ketogenic diet were followed in this observational study. Fecal and serum samples were collected at baseline and three months after dietary intervention.

**Findings:**

We identified both gut microbial and inflammatory changes during treatment. KD had a general anti-inflammatory effect. Novel bioinformatics and machine learning approaches identified signatures of specific Bifidobacteria and TNF (tumor necrosis factor) associated with responders before starting KD. During KD, taxonomic and inflammatory profiles between responders and non-responders were more similar than at baseline.

**Interpretation:**

Our results suggest that children with drug-resistant epilepsy are more likely to benefit from KD treatment when specific Bifidobacteria and TNF are elevated. We here present a novel signature of interaction of the gut microbiota and the immune system associated with anti-epileptic response to KD treatment. This signature could be used as a prognostic biomarker to identify potential responders to KD before starting treatment. Our findings may also contribute to the development of new anti-seizure therapies by targeting specific components of the gut microbiota.

**Funding:**

This study was supported by the Swedish Brain Foundation, Margarethahemmet Society, Stiftelsen Sunnerdahls Handikappfond, Linnea & Josef Carlssons Foundation, and The McCormick Genomic & Proteomic Center.

**Research in context**

**Evidence before this study**

Over the past few years, the role of the gut microbiota has been increasingly appreciated in neurological disorders, including epilepsy. It has been shown that the composition of the gut microbiota differs in patients with epilepsy from healthy controls and that the ketogenic diet (KD), an over 100-year-old alternative anti-seizure treatment, significantly changes both the taxonomic and functional composition of the gut microbiota. A role of inflammation in epilepsy has also been widely demonstrated in both patients and animal models.

**Added value of this study**

We investigated associations between the gut microbiota and systemic inflammation in children with drug-resistant epilepsy. We identified specific Bifidobacterial species (*B. longum* and *B. breve*) that associated with TNF (tumor necrosis factor). TNF, a pro-inflammatory cytokine, has previously been linked to epilepsy. Both, *B. longum/breve* and TNF levels were higher in patients that subsequently started ketogenic diet treatment and responded with decreased seizure frequency as compared to those who did not respond. Previous *in vitro* studies demonstrated a direct interaction of Bifidobacteria with TNF and thus support our results and our hypothesis that Bifidobacteria may interact with the immune system via TNF, and this might influence seizure threshold.

**Implications of all the available evidence**

Integrative machine learning models identified both *B. longum/breve* and TNF amongst the most important variables to distinguish responders from non-responders before starting the ketogenic diet. *B. longum/breve* and TNF might thus serve as biomarkers to identify potential treatment responders in the clinics. In addition, we may have identified a mechanism by which gut microbes contribute to seizures and targeted manipulation of the gut microbial composition might represent a novel anti-seizure treatment strategy. However, larger studies need to be performed to confirm our findings.

Alt-text: Unlabelled box

## Introduction

Epilepsy is a neurological disease that affects both children and adults. Although an increasing number of etiologies (e.g. structural, metabolic, or infectious) have been identified, the cause of epilepsy still remains unclear in many patients.^1^ Furthermore, as almost a third of epilepsy patients do not achieve adequate seizure control in spite of drug treatment, current strategies are clearly insufficient.^2^ Importantly, most available therapies are directed at symptomatic epilepsy management rather than targeting the pathogenic mechanisms of disease and the term “anti-epileptic drugs” has therefore been suggested to be altered to “anti-seizure medications”.^3^ Identifying pathophysiological mechanisms underlying the development (i.e. the epileptogenesis) and the sustained activity of epilepsy may offer new targets for pharmacological interventions in treating patients with intractable epilepsy and even target the development of disease *per se*. A role of inflammatory processes in both the epileptogenesis and sustainment of epilepsy has been demonstrated in both human and experimentally acquired epilepsies (reviewed in^3^), and recently also in genetic epilepsies.^4^ The relationship between inflammation and epilepsy is thus reciprocal with inflammation contributing to seizure precipitation and seizures inducing inflammation.

For children with refractory, i.e., pharmaco-resistant epilepsy, ketogenic dietary therapies, including classic ketogenic diet (KD), (Modified Atkins Diet (MAD) and Medium Chain Triglycerides (MCT) are well-established, non-pharmacological treatment alternatives that have been used for many years.^5^ KD is a high-fat, sufficient-protein and very low-carbohydrate dietary therapy. Randomized controlled studies have shown >50% seizure reduction of KD in up to one half of children with refractory epilepsy.^6^^,^^7^

During KD, as intake of carbohydrates is limited and intake of fats is drastically increased, the body induces ketosis with production of ketone bodies (β-hydroxybutyrate, acetoacetate and acetone) which are used as an alternative energy substrate for ATP production in cells. This metabolic shift induces many biochemical, metabolic and hormonal changes that may contribute to decreased neuronal excitability and seizure reduction. However, the underlying mechanisms of action of KD are still not clear. Neither is it clear, why some patients respond to the treatment with less seizures while other do not. Several potential hypotheses have been proposed. These include enhanced GABA-mediated inhibition, inhibitory actions of polyunsaturated fatty acids on ion channels, elevated ATP levels which convert to the inhibitory mediator adenosine, as well as increased mitochondrial biogenesis and reduced oxidative stress.^8^ Recently, other possible mechanisms of action have been proposed. These include anti-inflammatory actions, epigenetic changes, and changes in the gut microbial composition.^9^

The aim of this study was to analyse changes in the fecal microbiota and levels of blood inflammation markers after three months on KD treatment in a cohort of children with pharmaco-resistant epilepsy. Using state-of-the-art bioinformatics and machine learning methods we identified specific signatures of correlated microbial and inflammatory signals related to the anti-seizure effect of the diet.

## Methods

### Patients cohort description

Here, we present a prospective observational study on the composition of the fecal microbiota and the levels of serum cytokines in relation to the ketogenic diet and its anti-seizure effect in children with refractory epilepsy treated at the Astrid Lindgren´s Children´s Hospital, Karolinska University Hospital, Stockholm, Sweden. After a clinical decision was made to start the KD for treatment of refractory epilepsy, patients were asked to participate in the present study and were enrolled consecutively. The inclusion criteria were age older than 1.5 years and younger than 18; pharmaco-resistant epilepsy not accessible to epilepsy surgery, no medical contraindications to treatment with KD, no antibiotics or probiotics treatment within three months before sampling, and informed consent for participation in the study by a legal guardian.

In total, 28 children starting KD treatment participated. Twelve patients were included in our previously published pilot study.^11^ Two fecal samples (one before and one during treatment) were obtained from 27 children. Serum samples from both time points were available from 14 children. Three children only provided a serum sample at one time point. These were included in the unpaired analyses as described below. Demographics on the patient cohort and patients included in the sub studies are shown in Table 1. The age at KD initiation and first feces and blood sampling in the study group was 7.7 ± 4.6 (mean ± SD) years; range 1.9–17.8 years. The gender distribution was 11 boys and 17 girls. Their epilepsy onset was early with first seizure appearing at the age of 1.7 ± 2.7 (mean ± SD) years; range 0.1–12.7. The majority had generalized epilepsy, 22 children, versus focal epilepsy in six. Classification of seizure types was made according to the revised classification and terminology presented by the International League Against Epilepsy (ILAE).^10^ As seen in Table 1 most of the patients had more than one seizure type. The most common seizure types were tonic and generalized tonic-clonic. The etiology and epilepsy syndrome were classified according to the ILAE classification from 2017.^1^ The etiology was structural in ten children, genetic in twelve and in the remaining six it was unknown. Among the patients with structural lesions a genetic basis was found in three of these patients. An epileptic syndrome could be identified in nine children, with West syndrome in eight cases and Dravet syndrome in one.

**Table 1:** Demographics of the study populations. All patients included are presented as well as the patients in the sub studies.

Demographics	**_**	All patients	Fecal microbiome study	Blood Inflammation	Microbiome and inflammation
(Pat no.)		(*n* = 28)	(*n* = 27)	markers (*n* = 14)	markers (*n* = 17)
Age at KD start	mean ± SD (range)	7.7 ± 4.6 (1.9–17.8)	7.6 ± 4.8 (1.9–17.8)	9.3 ± 4.6 (2.8–17.8)	9.0 ± 4.5 (2.8–17.8)
Gender	male/female	11/17	10/17	5/9	5/12
Age at epilepsy onset	mean ± SD (range)	1.7 ± 2.7 (0.1–12.7)	1.7 ± 2.7 (0.1–12.7)	2.0 ± 3.5 (0.1–12.7)	2.1 ± 3.3 (0.1–12.7)
Type of epilepsy	gen/focal	22/6	21/6	10/4	12/5
Etiology					
	structural	10	10	5	5
	genetic	12	11	8	10
	unknown	6	6	1	2
No. of seizure types	mean ± SD (range)	1.9 ± 0.8 (1–4)	1,8 ± 0.6 (1–3)	1.8 ± 0.9 (1–4)	1.6 ± 0.7 (1–3)
Seizure types					
	focal w imp aware	9	9	5	6
	epileptic spasms	4	4	1	1
	tonic	11	11	4	5
	myoclonic	8	7	3	4
	generalized ton–clon	11	10	6	7
	abs	6	5	4	5
No. previous ASMs	mean ± SD (range)	6.6 ± 2.2 (3–12)	6.6 ± 2.3 (3–12)	6.6 ± 2.4 (3–11)	6.8 ± 2.7 (3–12)
No. concomitant ASMs	mean ± SD (range)	2.3 ± 1.1 (1–4)	2.2 ± 1.0 (1–4)	2.4 ± 1.2 (1–4)	2.2 ± 1.1 (1–4)
Comorbidity					
	Intellectual disability	25/28	24/27	12/14	15/17
	Motor dysfunction	16/28	16/27	8/14	10/17
Ratio KD at 3 mo.					
	2:1	1	1	0	1
	3:1	8	7	4	5
	3.5:1	11	11	5	6
	4:1	8	8	5	5
β-OHB at 3 mo.	mean ± SD (range)	4.2 ± 1.6 (0.8–6.6)	4.1 ± 1.5 (0.8–6.1)	4.3 ± 1.9 (0.8–6.6)	4.2 ± 1.8 (0.8–6.1)
Seizure response 3 mo.	no. (%)	16/28 (57%)	16/27 (59%)	7/14 (50%)	9/17 (53%)

Abbreviations: gen, generalized; no., number; focal w imp aware, focal onset with impaired awareness; ton-clon, tonic-clonic; abs, absences; ASMs, anti-seizure medications; mo., months; β-OHB, β-hydroxybutyric acid.

The children were all pharmaco-resistant and had previously been on trials with several anti-seizure medications (ASMs) with a mean of 6.6 ± 2.2 SD (range 3–12). At time of KD initiation and first sampling, the patients were all on daily ASM treatment with mean 2.3 ± 1.1 SD (range 1–4). The most commonly used ASMs were valproate (*n* = 14), topiramate (*n* = 8), and clobazam (*n* = 8). At three months on KD, when follow-up of seizure response and second sampling of fecal microbiota and blood was obtained, all 28 patients had the same ASMs as at KD start and the dosing was the same except in two patients on valproate (reduction 25–37%) and three patients on topiramate (reduction 20–25%) due to side effects.

Comorbidities were common. Intellectual disability was found in 25 out of 28 patients, ranging from mild to severe. In 24 of these children, a formal testing by a trained psychologist had been done using the standardized test battery best suited for the child, often Wechsler Intelligence Scale (WIPSY/WISC), Nepsy, Bailey and/or Wineland Neuropsychiatric comorbidities, as autism spectrum disorder, was also common, in 20 out of the 28 children. Twelve children had a gastrostomy i.e. a tube inserted through the abdomen and into the stomach due to difficulties in swallowing (dysphagia) or refusing to eat enough (risk of stunting). However, the majority of these children managed to take half or more of their food orally.

### Study design

In line with our previously published pilot study^11^ the design was as follows:

The efficacy of the KD was evaluated based on seizure response. Seizure frequency was calculated from seizure calendars routinely used in our clinic. Here, parents and other caregivers daily recorded the number and type(s) of seizures. The mean seizure frequency the month before initiation of the KD was calculated as baseline and was compared to the mean frequency during the month preceding follow-up visit at three months on diet. Children with ≥50% seizure reduction were classified as responders and those with < 50% reduction as non-responders.

In this study we used the classical KD and followed a standardized protocol, which is a slightly modified version of the protocol of the Johns Hopkins Hospital.^12^ A dietitian, specially trained to carry out KD treatment, calculated the total calorie level per day and composition of meals and supplements for each child. The calculations of the total calorie amount and the energy percentage derived from fats, proteins and carbohydrates was based on a 2-day diary kept by the parents before admission in which they recorded all food consumed by the child. The caloric level was then modified to the recommended calorie amount according to age, weight as well as to the expected level of physical activity. Fasting or restriction of calories or fluids were not utilized. A minimum of 1g/kg body weight per day of protein was included. The children were supplemented with multivitamins and minerals, including potassium, calcium, magnesium, zinc, selenium, and with 100 mg/kg/day of carnitine. Potassium was given as citrate to reduce the risk of kidney stones.

For the initiation of KD, the child was hospitalized for about four days for surveillance and educational purposes of caregivers. Before the diet start, a blood sample was obtained in the morning before breakfast for analyses of glucose, the ketone β-hydroxybutyric acid (β-OHB) and inflammatory markers. During the hospital stay KD meals were introduced. The diet was monitored by clinical examinations and daily blood levels of β-OHB, glucose, and acid-base balance to avoid hypoglycemia and acidosis. KD was usually started on a ratio of 2:1. This ratio was increased once a week by 0.5. Blood was sampled to monitor ketones, glucose and acid-base balance after increases in ratio. An optimal ratio for the individual was normally reached within 3–6 weeks. This ratio was kept constant until three months after start when KD was evaluated concerning efficacy and the second fecal and blood samples were taken.

All children were taking ASMs during KD. The rationale was trying to avoid ASM dose changes before evaluation of KD at three months to minimize confounding factors which was achieved in the majority of cases (see Methods, Patients cohort description).

### Fecal sample collection, transport and storage

Fecal samples were obtained by swab from the diaper or toilet paper by a parent using a sterile FLOQSwab™ (Copan). The first sample was taken at home the day before starting KD or in the morning of the day of starting. The second sampling at three months on diet was collected at home and the swabs were kept in a refrigerator for a few hours until transported to the hospital on ice and immediately stored at −70 °C.

### Serum collection and storage

The first blood sample was obtained within a week before starting KD treatment and the second after three months on diet at time of follow-up visit. Both samplings were performed in the morning before breakfast and before intake of ASMs. The samples were collected in standard EDTA tubes by trained nurses at our Epilepsy Outpatient Clinic. Blood samples were cold-centrifuged for 30 min and the supernatant was collected in Eppendorf tubes, which were immediately frozen and stored at –70 °C. All samples were analysed for inflammation markers within the same time frame.

### Ethics

The study was approved by the local Ethics Committee (Dnr 2014/1177-32). Informed consent was given by the legal guardians of the children and, whenever possible, the children themselves. This study was conducted in accordance with relevant guidelines and procedures.

### Fecal samples processing

Fecal samples were processed identically to the samples from our pilot cohort published as Lindefeldt et al.^11^ In short, total nucleic acids were extracted from fecal samples using the PowerMicrobiome™ RNA Isolation Kit (Qiagen) according to the manufacturer's instructions. Following incubation with 10 ng PureLink RNase A (Invitrogen) at 37 °C for 30 min, sodium acetate (3 M, pH 6.8) isopropanol were added for 10 min on ice. DNA was pelleted and washed in ethanol (70% v/v), centrifuged, and dried at room temperature. Finally, purified DNA was re-dissolved in RNase-free water and stored at −80 °C.

DNA was quantified with the Qubit dsDNA HS Assay Kit (Thermo Fisher Scientific). 250 ng DNA was sheared in the Covaris® S2 instrument (Covaris, Inc.) to an insert size of approximately 650 bp. Fifty nanograms of sheared DNA was used for preparation of sequencing libraries with the ThruPLEX® DNA-seq Kit (Rubicon Genomics) according to the manufacturer's instructions using provided primers with ten cycles for amplification. Primer sequences were as follows:

5’:AATGATACGGCGACCACCGAGATCTACACNNNNNNNNACACTCTTTCCCTACACGACGCTCTTCCGATCT with NNNNNNNN being a TruSeq HT i5 index and 3’:GTTCGTCTTCTGCCGTATGCTCTANNNNNNNNCACTGACCTCAAGTCTGCACACGAGAAGGCTAGA with NNNNNNNN being a TruSeq HT i7 index.

PCR products were purified on the MBS Magnatrix 1200 automated workstation (NorDiag) with Dynabeads® MyOne carboxylic acid beads (Thermo Fisher Scientific). Purity of the samples and insert size distribution was evaluated using the High Sensitivity DNA Kit on the Agilent 2100 Bioanalyzer instrument (Agilent Technologies). DNA libraries were quantified with the Qubit dsDNA HS Assay Kit (Thermo Fisher Scientific) and equimolar concentrations of 12 samples were pooled and further purified using Agencourt AMPure XP (Beckman Coulter, Inc.). Library pools with a final concentration of 10 nM DNA were submitted to one flow cell per pool and sequenced using the MiSeq V3 chemistry (Illumina).

BBDuk^13^^,^^14^ was used to quality-filter and trim raw reads with the following parameters: ktrim = r *k* = 23 mink = 6 hdist = 1 qtrim = rl trimq = 20 minlength = 70 tpe tbo. The quality of the sequences before and after was visually inspected using MultiQC.64

### Taxonomic profiling of fecal microbiota

Taxonomic composition profiling using FASTQ output files was performed using previously described methods^15^, ^16^, ^17^, ^18^ resulting in a table of bacterial composition with relative abundance calculations (Supplementary Table 1). Paired end FASTQ files were assessed using the QC function in HIVE, that includes Lengthwise Positional Count and Quality Positional Count graphs. As an additional pre-filtering QC step, all metagenomics samples were run against the human reference genome GRCh38.p2 using HIVE-hexagon to filter-out any host (human) DNA (Supplementary Table 2). All unaligned reads were then analysed with CensuScope against a version of the NCBI Nucleotide Database, where synthetic and other ambiguous sequences are removed^19^^,^^20^ (CensuScope parameters are available in Supplementary Table 3). The CensuScope output from all metagenomics samples were curated to create a unique list of GenBank accessions,^21^ which taxonomically accounts for all known organisms within the samples. These data were used to create a reference database for the final alignment step using HIVE-hexagon (HIVE-hexagon parameters available in Supplementary Table 4).

HIVE-hexagon produces a “hit list” of organisms and relative abundances (RA) for each input sample were calculated. QA/QC methods incorporated to build the final data set from the individual hit lists include: a) removing all ambiguous hits e.g. scaffolds, etc. - keeping only complete genomes or complete sequences from bacteria; b) condensing and curating multiple strains/isolates under one organism and proteome cluster using the Protein Informatics Resource (PIR) Reference Proteomes database at a 75% cut-off.^22^^,^^23^ The original dataset comprised 488 organisms, many of them being multiple strains/isolates of a species. The final data set was condensed to 216 organisms using the methods described above. Once the evaluation was complete, the bacterial RAs were summed under the chosen bacteria. The final data set contained the RAs and taxonomic microbiome profile of all input samples, (Suppl. Table 1). This master data set and specific patient metadata was then used to make subsequent datasets to serve as input files for specific questions posed in the machine learning (ML) analysis portion of our study.

### Machine learning analysis of the metagenomic dataset

With the application of machine learning methods developed and previously described^15^ we aimed to capture microbial ‘signals’ answering the following questions:

Box 1. **Six key questions were outlined to identify associations between gut microbiome profiles, KD treatment and anti-seizure responses.**

**Table:** 

**Q1)** Is there enough signal to classify whether a patient is on a KD or not? **Q2)** Which bacteria are important for distinguishing whether a patient is on a KD or not? **Q3)** Is there enough signal to distinguish the gut microbiome profiles of responders (R) from non-responders (NR) before starting a KD? **Q4)** Are there specific organisms that are important for distinguishing R from NR before treatment? **Q5)** Is there enough signal to distinguish the gut microbiome profiles of R from NR three months after starting KD? **Q6)** Are there specific organisms that are important for distinguishing R from NR three months after starting KD?

Alt-text: Unlabelled box

As aforementioned, specific datasets were curated and derived from the master dataset to approach each question. Each input data set and question was analysed in MATLAB^24^ using the Statistics and Machine Learning Toolbox. All available algorithms (Suppl. Figure 1) were trained on the respective datasets for Q1, Q3, and Q5, with specific parameters selected in the Classification Learner for each (Suppl. Table 5). The model selection was based on estimated accuracy, ROC/AUC, and confusion matrix results (Suppl. Figure 2A–C). The Predictor importance of each model was calculated using out-of-bag permutation techniques run 10,000 times and averaged,^25^ resulting in the identification of the top 15 bacteria and their relative contribution to the respective model accuracy.

### Analysis of serum inflammation biomarkers

Serum proteins were measured using the Olink® INFLAMMATION panel* (Olink Proteomics AB, Uppsala, Sweden) according to the manufacturer's instructions. The Proximity Extension Assay (PEA) technology used for the Olink protocol has been well described,^26^ and enables 92 analytes to be analysed simultaneously, using 1 µL of each sample. In brief, pairs of oligonucleotide-labelled antibody probes bind to their targeted protein, and if the two probes are brought in close proximity the oligonucleotides will hybridize in a pair-wise manner. The addition of a DNA polymerase leads to a proximity-dependent DNA polymerization event, generating a unique PCR target sequence. The resulting DNA sequence is subsequently detected and quantified using a microfluidic real-time PCR instrument (Biomark HD, Fluidigm). Data is then quality controlled and normalized using an internal extension control and an inter-plate control, to adjust for intra- and inter-run variation. The final assay read-out is presented in Normalized Protein eXpression (NPX) values, which is an arbitrary unit on a log2-scale where a high value corresponds to a higher protein expression. All assay validation data (detection limits, intra- and inter-assay precision data, etc.) are available on the manufacturer's website (www.olink.com).

*Previously branded as Olink® Multiplex panels

### Statistical analyses

Differences in macronutrient intake between responders and non-responders were tested using a t-test for independent variables in Statistca (version 8). All other statistical analyses and visualizations were performed in R version 4.0.1 (2020-06-06). Taxa with a total abundance of <0.05% in the metagenomic dataset were filtered out. We used centered log ratio (clr)-transformed relative abundance values for statistical analysis of the metagenomics dataset. Statistical comparisons were performed using Welch's t-test, for correlation analysis, Spearman's correlation was applied.

Partial least squares discriminant analysis (PLS-DA) was performed using DIABLO (Data Integration  Analysis for Biomarker discovery using Latent variable approaches for Omics studies)^27^^,^^28^ within the mixOmics R package version 6.15.33 (2021-01-27). For the omics integration models, a design based on the correlation values of component one of a PLS analysis for taxa and inflammation markers was used. The number of components and the type of distance to be included in the tuning of each model was selected based on the lowest error rate achieved by running the “perf” function. A range of variables for both taxa and inflammation markers was tested in tune.block.splsda using leave-one-out cross-validation. Correlation coefficients between important variables of each dataset were calculated according to Ignacio González et al.^29^ and correlations larger than the indicated cut-off were visualized in circos plots.

### Data availability

Raw sequencing data have been submitted to the European Nucleotide Archive under study accession number PRJEB28847 (pilot cohort) and PRJEB49521 (extended cohort).

### Role of funders

Our funding sources did not have any influence on study design, data collection, analyses or interpretation. Neither did they influence the context of the manuscript.

## Results

The study included 28 children with pharmaco-resistant epilepsy treated with the KD. For inclusion and exclusion criteria of the patients, demographics, and treatment details, see Methods and Table 1. Fecal samples as well as blood samples were collected at two time points, before and three months after initiation of KD. At the second time point the mean ketogenic ratio for the 28 patients was 3.4 ± 0.5 (mean ± SD) and the ratio was 4:1 in eight children, 3.5:1 in eleven, 3:1 in eight and 2:1 in one. Before diet start, the baseline levels of β-hydroxybutyric acid (β-OHB) were 0.4 ± 0.8 mmol/L (mean ± SD), median 0.2 and range 0.1–4.2 (*n* = 25). At three months on KD, β-OHB was 4.2 ± 1.6 mmol/L (mean ± SD), median 4.4 with a range of 0.8–6.6 mmol/l (*n* = 28).

The seizure response in relation to the diet is shown in Table 1. The number of responders, ≥50% seizure reduction, at three months on diet was 16 out of 28 patients (57%). Two patients were seizure free. The mean daily seizure frequency at baseline was 5.6 ± SD 6.0 (range 0.1–20) and during KD 2.4 ± SD 4.0 (range 0–18). The relative changes in seizure frequency during diet versus before diet showed a mean reduction of 44.0% ± SD 38.9 (median 54.0%; range reduction 100%- increase 19.0%).

The children that were considered responders with a ≥50% seizure reduction (*n* = 16) had a mean seizure reduction of 73.9% ± SD 17.8% (median 68.3%; range 100–51%) and those considered non-responders (*n* = 12) had mean reduction of 4% ± SD 15.2% (median 2%; range reduction 40%- increase 19%). Among the non-responders two children had a seizure increase, 18% and 19%, respectively.

The baseline energy percentage of intake of fats, proteins and carbohydrates before diet start could be calculated in 23/28 children based on complete 2-day food diaries. In seizure responders, the intake in energy percentage of fats, proteins and carbohydrates was 37.3%, 16.7% and 46.0% and in non-responders 41.2%, 16.6% and 42.2%, respectively, prior to dietary intervention. The ratio of fats to proteins and carbohydrates were 0.28:1 in responders and 0.30:1 in non-responders. No statistically significant differences were found in relation to response.

### The composition of the gut microbiota changes during the ketogenic diet

**Figure 1:**
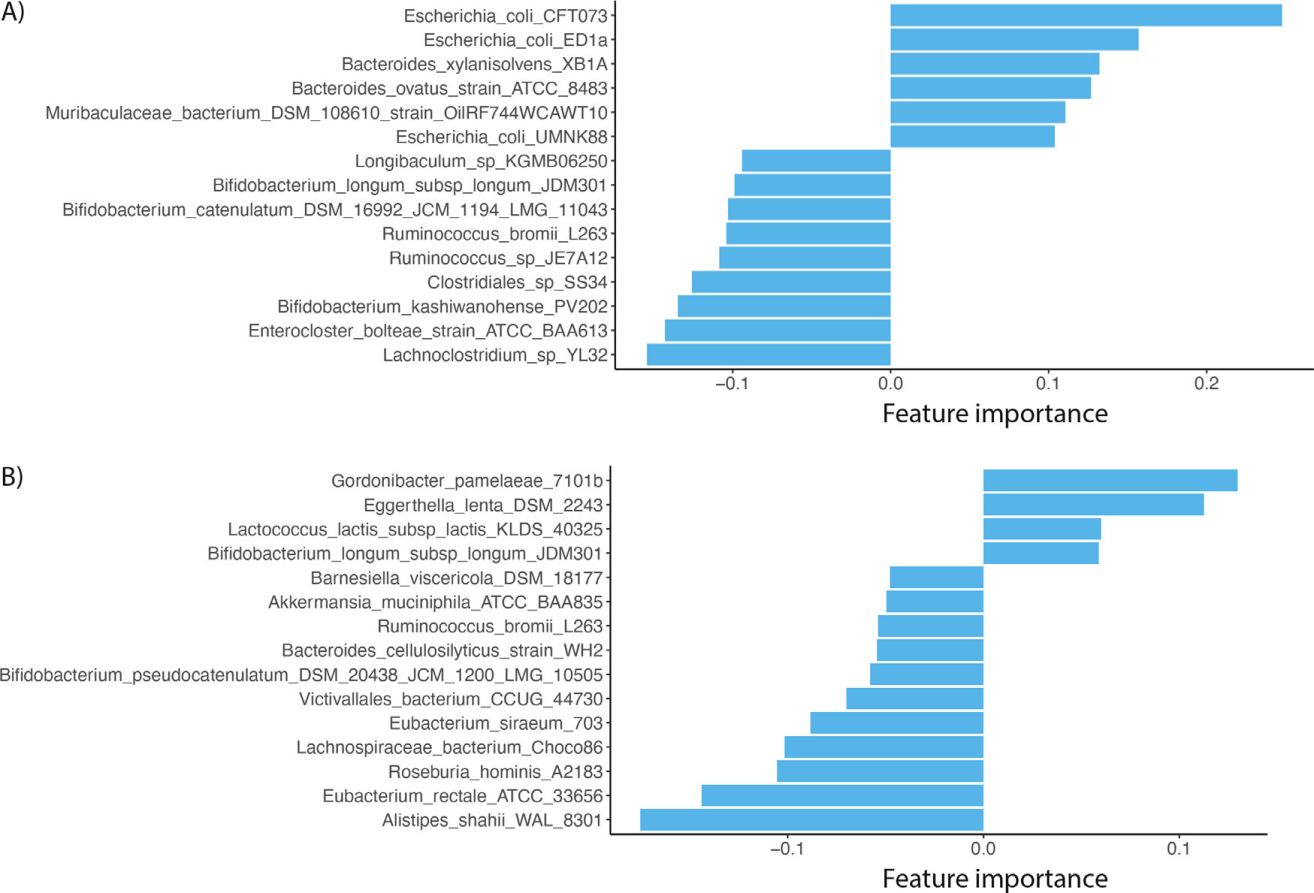
Bacterial taxa contributing to the final models ranked by feature importance. A) Comparing all patients before vs during KD and B) responders vs. non-responders before KD. A) showing feature importance of taxa positively or negatively associated with KD and B) showing feature importance of taxa positively or negatively associated with anti-epileptic response to KD.

The Classification Learner results for Q1(Is there enough signal to classify whether a patient is on a KD, or not? Box 1 in Methods section) identified the models fine, medium, and coarse trees (decision trees),^30^^,^^31^ to have the highest accuracy compared to all available algorithmic models (Suppl. Figure 1). Fine, medium, and coarse tree performed equally across all plots, at 80.9% estimated classification accuracy. Model 1.3 coarse tree was selected to avoid overfitting, as it is preferable to select a model of lower flexibility that provides sufficient accuracy. Coarse tree's flexibility is the lowest of the decision trees and has fewer ‘leaves’ to make coarse distinctions between classes (maximum number of splits is 4). Predictive accuracy of the coarse tree model was further evaluated through the confusion matrices, ROC, and AUC (Suppl. Figure 2A). The top 15 bacterial species contributing to the observable overall accuracy of the coarse decision tree model^32^ are shown in Figure 1A (Q2 results), and confirm our results previously published.^11^ The Classification Learner results for Q3 (Is there enough signal to distinguish the gut microbiome profiles of responders from non-responders before starting a KD?), identified the model Ensemble Subspace k-nearest neighbours (kNN)^33^ to have the highest estimated accuracy at 71.4%, compared with all available algorithmic models. Ensemble methods combine multiple classifiers, which provides a marked improvement in predictive accuracy and overall performance, particularly when less delineated characteristics exit in the dataset.^34^ Additionally, the kNN algorithm is known for its simplicity and efficiency. Predictive accuracy of the Subspace KNN model was further evaluated through the confusion matrices, ROC, and AUC (Suppl. Figure 2B). The top 15 bacteria ranked by contribution of observable overall accuracy of the model are shown in Figure 1B (Q4 results). The Classification Learner results for Q5 (Is there enough signal to distinguish the gut microbiome profiles of responders from non-responders three months after starting KD?) were indiscriminate. The model with the best estimated classification accuracy was ensemble boosted trees, with an estimated accuracy of 58.3%, which is close to the actual response rate of 58% in this dataset (Suppl. Figure 1C). For Q5, the AUC/ROC was unable to be generated in MATLAB, and the predictor importance which would have been included as results for Q6, were not included to avoid providing misleading information. All predictive measures were computed out of sample.

Results for all six exploratory questions should be viewed as preliminary findings to help guide and strengthen future hypotheses, rather than concrete answers. Here, through Q1-Q6, we demonstrate the concept of applying ML analysis in conjunction with a multifactorial approach to build a well-rounded picture. Our machine learning analysis of the metagenomics dataset suggests there is a distinct signal allowing the delineation of an individual on KD or not, with an estimated predictive accuracy of 80.9% with coarse decision trees. Important bacteria contributing to this signal include several *E. coli* subspecies and Bifidobacterial species, but also Bacteroides and Lachnoclostridium. Responders could be distinguished from non-responders with an accuracy of 71.4% using a KNN-model. Here, *Gordonibacter pamelaeae, Eggerthella lentha, Lactococcus lactis* and *Bifidobacterium longum susp. longum* were associated with an anti-seizure response to KD whereas other bacteria, including *Alistipes shahii* and *Eubacterium rectale* were associated with a lack of response. We were not able to build a model that could distinguish between responders and non-responders during KD treatment, indicating the absence of useful discriminative features in the fecal microbiota at this time point.

### The ketogenic diet impacts several inflammation biomarkers in patient serum

**Figure 2:**
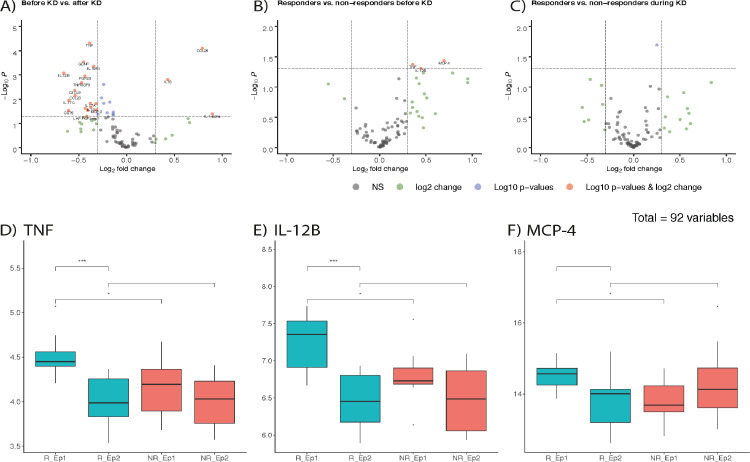
Inflammation profiles in children with refractory epilepsy and changes during the ketogenic diet. Volcano plots showing differences in inflammation markers (A) due to KD treatment or (B) comparing responders to non-responders before starting KD, and (C) after three months of treatment. Blue dots depict significant changes (*p* < 0.05), green dots represent markers with a log2 fold change of >0.3, while red dots fulfil both criteria and black dots none. D-F) show boxplots of TNF, IL-12B and MCP-4, respectively, for responders before KD (R_Ep1) and during KD (R_Ep2) as well as for non-responders before KD (NR_Ep1) and during KD (NR_Ep2). A centre line represents the median; box limits are upper and lower quartiles; whiskers show 1.5 × interquartile range; and points depict outliers. Asterisks indicate significance of Welch's t-test as follows: *** *p* < 0.001, ** *p* < 0.01, *p < 0.05.

We obtained serum samples from a subgroup of patients (*n* = 14) before starting KD and three months into treatment. Using the Olink® inflammation panel, we profiled systemic inflammation using 92 inflammation-related protein biomarkers. We found that 29 of these significantly changed during dietary intervention (*p* < 0.05, paired Welch's t-test). Out of these, only three increased, namely CCL25, IL-18, and IL-1 alpha, while 26 decreased in expression, including IL-17A, IL-17C, TNF, IL-12B, IL-18R1, and GDNF (Figure 2A).

Before starting KD, inflammation was slightly higher in those who responded to the dietary intervention with >50% seizure reduction (Figure 2B). Many of those increased inflammation markers were not significantly higher, possibly due in part to the small number of individuals. However, TNF, IL-12B, and MCP-4 were significantly increased in responders before KD compared to non-responders before KD. Of note, both TNF and IL-12B significantly decreased during KD while MCP-4 did not (Figure 2D–F). Comparing the inflammation profiles of responders to non-responders during KD there was no clear difference (Figure 2C). Only one inflammation marker (IL-20A) was significantly different with a very small fold change between responders and non-responders during KD.

### Multivariate PLS-DA identified associations between inflammation markers and gut microbes

**Figure 3:**
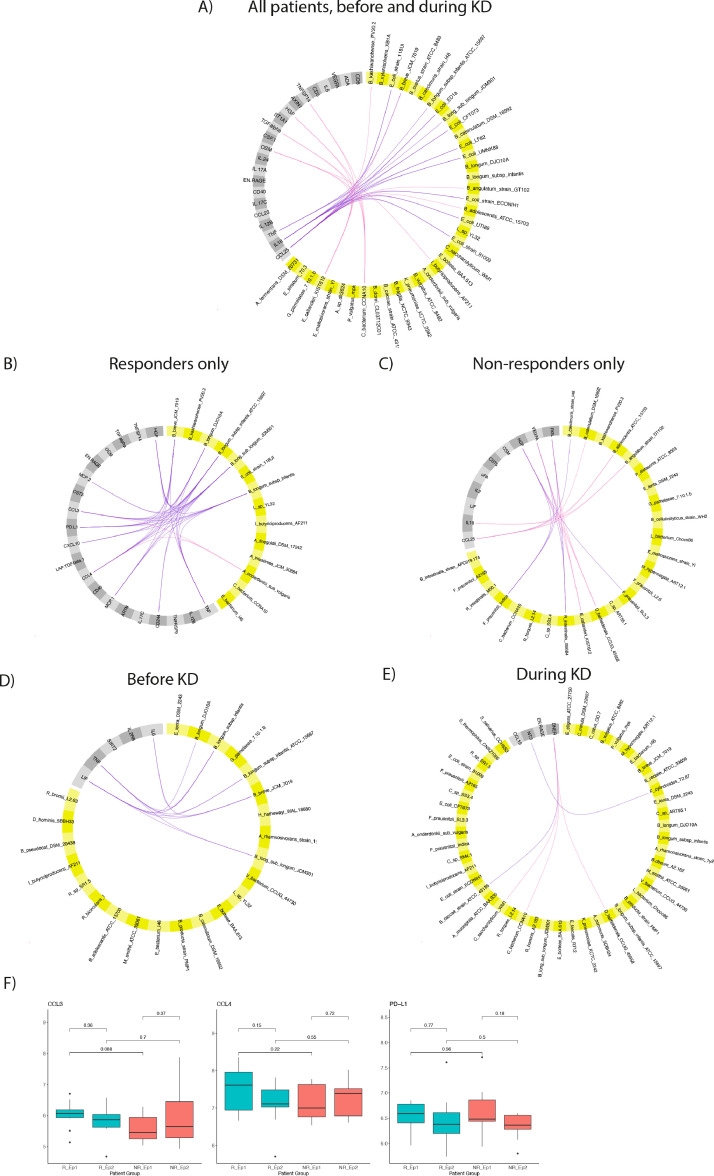
Multivariate PLS-DA of associations between inflammation markers and gut microbes. In A) PLS-DA for all patients with metagenomics and inflammation profiles was performed using dietary treatment (before vs. during) as the discriminating factor. The contributing metagenomic variables are depicted in yellow and the contributing inflammation variables are grey. Strong positive correlations between variables of each dataset are shown as purple lines and strong negative correlations are pink. For visualization purposes only the top correlations were chosen in each figure. For A) a cut-off of 0.6 was chosen, while 0.7 was used in Figure B-E. In B) the same analysis was performed as in A) but for responders only and in C) for non-responders only. Circos plots of PLS-DA models comparing responders to non-responders before KD and during are shown in D) and E), respectively. In F) boxplots of levels of CCL3, CCL4 and PD-L1 are shown for responders before KD (R_Ep1) and during KD (R_Ep2) as well as for non-responders before KD (NR_Ep1) and during KD (NR_Ep2) and p-values from t-tests for each comparison were added above. Boxplots show a centre line representing the median; box limits are upper and lower quartiles; whiskers show 1.5 × interquartile range; and points depict outliers.

We used a supervised machine learning approach to build models integrating both gut microbial profiles and inflammation profiles. We chose DIABLO^15^ to model changes in both -omics datasets during KD. DIABLO applies PLS-DA to identify common information across different data sets by selection of a subset of features and thorough cross-validation. We first built a model with all inflammation markers and complete taxonomic profiles from all patients that had both datasets for either time point. The resulting model had an accuracy of 0.84 using the first component with 23 taxa and 6 inflammation peptides (Table 2). Adding the second component increased this accuracy to 0.93 by adding another 23 taxa and 17 inflammation peptides. In component one, *E. coli* strains were dominating the taxa associated with KD treatment, while Bifidobacteria were the dominating taxa associated with samples before treatment, i.e. decreasing during treatment. Again, these are taxa previously identified in our pilot study and our ML model herein. Five out of the six inflammation variables contributing to component one were also amongst the most significant peptides identified in our paired univariate analysis, i.e. CCL25, IL-18, TNF, IL-12B and CCL23. Correlation coefficients were calculated and visualized in a circos plot (Figure 3A) where positive correlations between TNF and *Bifidobacteria* (*B. breve, B. longum subspecies infantis ATCC 15697 and B. longum subspecies JDM301*) were identified. We also found a positive correlation of several *E. coli* strains with both CCL25 and IL-18, as well as a negative correlation between CCL25 and other Bifidobacteria (*B. angulatum, B. adolescentis, B. kashiwanohense*) but also *B. breve*. The strongest associations identified in component two were between *Eubacterium callanderi* strain KIST612, *Clostridiales bacterium* CCNA10, *Intestinimonas butyriciproducens* strain AF211, and OSM, TGF- α, HGF and TNFSF14. Next, we used the same modeling approach to identify important taxa-inflammation signals for either responders only or non-responders only. These models did not achieve as low error rates as the model including all samples, likely due to smaller samples sizes (Table 2). Interestingly, the correlations identified amongst responders were different from the non-responders as shown in the circos plots (Figure 3B,C). In non-responders, the negative association between non-longum Bifidobacteria and CCL25/IL-18 was dominating, while the positive correlations between TNF and *B. longum*/*breve* were specifically recapitulated in the responders only. In the responders, the correlation coefficients of *B. longum/breve* were slightly higher for CCL3 and CCL4 than for TNF, followed by PD-L1. Univariate analysis of CCL3, CCL4 and PD-L1 did not show significant differences between responders and non-responders before or during KD in this small dataset (Figure 3F). However, a similar trend for CCL3 and CCL4 as for TNF was observed, i.e., the highest mean value was observed for responders before starting treatment. The final PLS-DA model for both responders and non-responders’ samples before KD only showed strong positive associations of four *B. longum* subspecies as well as *B. breve* with LIF, TNF and IL-6. (Figure 3D). All these correlations were responders-associated,and they were absent in responders vs. non-responders during KD (Figure 3E), where various taxa were positively or negatively correlated with DNER and NT-3. For visualization purposes, only the strongest correlations are depicted in Figure 3A–E, however, the full correlation matrices for each model can be found in Supplementary Tables 6–10.

**Table 2:** Overview of PLS-DA models integrating metagenomics and inflammation datasets.

Comparison	Component 1	Component 2	Component 3	Component 4
**All** before vs during KD	0.84	0.93*	0.93	not tested
	23/6	23/17	41/11	-
**Responders** before vs during KD	0.69*	0.56	0.69	0.69
	14/20	17/35	5/23	5/5
**Non-responders** before vs during KD	0.71	0.77*	0.79	0.86
	5/5	20/5	5/17	23/5
Non-responders vs responders **before KD**	0.79*	0.73	0.81	not tested
	23/5	26/5	6/38	-
Non-responders vs responders **during KD**	0.69	0.75*	0.75	not tested
	30/3	18/1	12/1	-

Grey rows: accuracy for each component of the final PLS-DA models.

White rows: number of variables in each component (taxa/inflammation).

⁎ Model chosen for circos plots.

**Figure 4:**
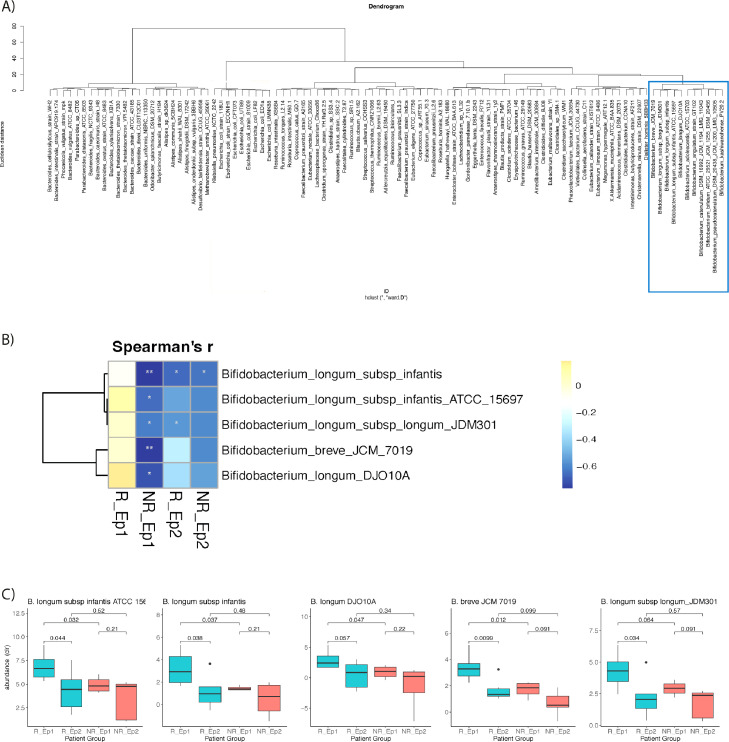
Responder-specific Bifidobacterium profiles. Hierarchical clustering of Pearson's correlations of Bifidobacteria in patients before starting KD is shown in (A). Spearman's correlation of members of the *B. longum* cluster with age are shown in B). Colour gradients in B) indicate the nature and strength of each correlation (Spearman's r), while Asterisks indicate significance as follows: *** *p* < 0.001, ** *p* < 0.01, * *p* < 0.05. Boxplots for each member of the *B. longum* cluster in children over eight years of age for responders before KD (R_Ep1) and during KD (R_Ep2) as well as for non-responders before KD (NR_Ep1) and during KD (NR_Ep2) with a centre line representing the median; box limits, are upper and lower quartiles; whiskers show 1.5 × interquartile range; and points depict outliers. Numbers above boxplots indicate p-values of Welch's t-test.

Although several Bifidobacteria species decrease during KD, certain species correlated with different inflammation markers. While *B. kashiwanohense* PV20-2, *B. angulatum* strain GT102, *B. adolescentis* ATCC 15703 and *B. breve* JCM 7019 showed significant negative correlations with CCL25. *B. breve* JCM 7019 and three *B. longum* strains showed significant positive correlations with TNF in the full dataset. Pearson correlation and hierarchical clustering of ward distances revealed that these Bifidobacteria were separated into two distinct clusters which we named “*B. longum* cluster” and “diverse Bifido cluster” (Figure 4A**)**. The clustering reflected the distinguished correlation patterns of Bifidobacteria with inflammation markers. Members of the *B. longum* cluster correlated positively with TNF, whereas members of the diverse Bifido cluster significantly negatively correlated with CCL25 (Figure 3**)**. Thus, these Bifidobacteria may exhibit different functions. Bifidobacteria, especially *B. breve* and *B. longum*, are typically associated with breastfeeding and an immature gut microbiota. They are more abundant during early life and decrease with age.^35^ No participant was breastfed during our study and the youngest was 1.9 years old. However, the decline of Bifidobacteria may occur during the first years of life. Our cohort had an age range of 1.9 to 17.8 years, the mean age of non-responders was 7.3 years before starting KD and for responders it was 8.0. We analysed whether the Bifidobacteria in our samples negatively correlated with age. We observed negative correlations of *B. longum* cluster members with age (either significantly or as a trend) in the non-responders both before starting KD and during KD (Figure 4B). In responders, however, this correlation was absent prior to KD treatment. After three months on KD, responders converted to moderate negative correlation of *B. longum* cluster members with age. At young age (below four years), responders and non-responders had comparable relative abundances of members of the *B. longum* cluster; however, the older the patients were, the more apparent the differences became. Responders exhibited higher relative *B. longum*/*breve* abundance compared to non-responders with the most pronounced differences above 15 years of age. Members of the diverse Bifidobacterium cluster did not significantly correlate with age before starting KD treatment (data not shown). Thus, responders kept Bifidobacteria associated with an immature gut microbiota over a longer time than non-responders or healthy children in other cohorts. This phenomenon was abrogated by KD treatment. In patients older than eight years almost all members of the *B. longum* cluster were significantly higher in responders than non-responders before KD (Figure 4C) while these differences were diminished during KD.

## Discussion

In a previously published pilot study, we identified *E. coli* and Bifidobacteria as the main taxa influenced by KD treatment of children with drug-resistant epilepsy.^11^ Here, we could confirm these signatures in a larger cohort. Albeit using a different approach for taxonomic profiling and analyses, including machine learning, these taxa were identified among others as important drivers of microbial changes during KD. Our new approach enabled higher resolution at species and strain level. Changes in relative abundance of several *E. coli* strains and various Bifidobacterial species were associated with the dietary intervention.

Recently, several studies investigating the gut microbial changes during KD in epilepsy patients have been published and several reviews have summarized these findings.^36^^,^^37^ Unfortunately, most studies were small in cohort size as KD is an expanding but still not common treatment option in epilepsy. These studies employed different designs with varying compositions of the diet and treatment lengths. Sample processing and analysis differed as well as demographics. Thus, the lack of consensual findings is not surprising. However, our findings are in agreement with several other studies. We find Bacteroides as important taxa associated with KD as previously demonstrated.^38^^,^^39^ In three independent cohorts comparing the gut microbiota of drug-resistant epilepsy patients and healthy controls, Bactereoides were decreased in epilepsy.^38^^,^^40^^,^^41^ Importantly, the decrease in Bifidobacteria during KD previously identified in our pilot study^11^ and by Xie et al.^38^ was recently confirmed by Gong et al.^42^ Unfortunately, the gut microbiota was analysed using 16S rRNA gene sequencing (V4 region) which does not provide species-level identification of Bifidobacteria. In addition, the genus Bifidobacteria was increased in drug-resistant epilepsy patients compared to healthy controls within the same age-range in one of the largest cohorts published until today.^43^ Lee *at al*.^41^ also observed a significant increase in the *Bifidobacterium longum* group in children with epilepsy compared to age-matched controls. During KD treatment of overweight or obese adults both a decrease in Bifidobacteria and an increase in *E. coli* was observed.^44^ Here, Bifidobacteria abundance was negatively associated with BHB levels and Th17 cells. Functional analysis by Lindefeldt et al.^11^ revealed a significant decrease in several microbial pathways of carbohydrate metabolism during KD associated with the decrease in Bifidobacterial abundances. As KD is usually deprived of fiber and Bifidobacteria thrive on dietary fiber,^45^ this finding is not surprising.

Our machine learning approach demonstrated that there was a discriminating microbial signature in fecal samples comparing patients before starting KD to after three months on KD. To some extent, we could also distinguish responders from non-responders at baseline based on their fecal microbes. However, the poor performance of the algorithms to delineate responders from non-responders after three months of KD indicates little differences in the gut microbial profiles during KD. Similar findings in the inflammation profiles suggest that responders have a different gut microbial and inflammation profile before treatment, which may contribute to seizure propensity.

Inflammation has been shown to play a role in both the origin of individual seizures as well as the epileptogenic process between seizures in both patients and in animal models.^46^, ^47^, ^48^, ^49^, ^50^, ^51^, ^52^ According to Meng et al., inflammation may be both the trigger and result of epilepsy. Thus, treatment strategies mitigating inflammation are worth exploring.^53^ Systemic inflammation can potentially disrupt the blood-brain barrier (BBB) enabling infiltration of peripheral toxic molecules and cytokine-producing immune cells and thus contribute to epileptogenesis.^54^ Protection of BBB integrity may diminish seizure occurrence.^55^

The ketogenic diet has been shown to possess anti-inflammatory properties.^56^, ^57^, ^58^ Previous studies on the effect of KD on systemic inflammation demonstrated changes in cytokines levels, e.g., decrease in TNF, IL-1ß and IL-17A.^56^^,^^59^ We observed an overall decrease in inflammation-related peptides during treatment, with few exceptions (Figure 2A). We were able to confirm the previously described decrease in TNF and IL-17A.

Only three of the 92 inflammation-associated peptides increased significantly during KD, namely IL-18, CCL25 and IL-1alpha. IL-18 has been proposed to help maintain a healthy gut microbiota and to protect from colitis likely involving induction of mucin production.^60^, ^61^, ^62^, ^63^ However, while IL-18 increases in serum during KD, systemic IL-18R1 (also known as IL-18Rα or IL-1R-related protein (IL-1Rrp)), its essential receptor,^64^ decreases significantly. IL-18-binding protein was not analysed. Thus, the role of IL-18 during KD may be confined to the gut but remains currently unclear. Under local inflammatory conditions, expression of CCL25 in the colon can be induced as observed in patients with ulcerative colitis (UC) where it correlated with disease severity.^65^ UC is commonly associated with increased abundance of various pathogenic *E. coli* strains in the gut^66^, ^67^, ^68^, ^69^ and a role for *E. coli* in CCL25 induction has been suggested.^70^ The increase of CCL25 in our cohort correlated with an increase in several *E. coli* strains during KD. Taken together, these findings may indicate *E. coli*-associated increase in colonic inflammation during KD but further studies are needed to evaluate the consequences of the potential risk for intestinal inflammation during KD treatment as previously discussed by Lindefeldt et al.^11^ We need to keep in mind that the methodologies used in this study only render relative abundance of fecal microbes. Thus, a relative increase in *E. coli* during KD might reflect a mere decrease of most gut microbes upon fiber-deprivation and not an actual increase in *E. coli*.

We found that responders to KD had significantly higher serum levels of both TNF and IL-12B than non-responders before starting treatment and these levels decreased significantly during three months of dietary intervention. In the brain, TNF^71^ can increase the number of glutamate receptors and decrease the number of GABA receptors via endocytosis.^72^ This leads to increased excitatory and decreased inhibitory synaptic strength. Increase of TNF due to neuronal insults might thus result in neuronal dysfunction and overexcitation potentially causing or exacerbating seizures. High levels of systemic TNF have been associated with epilepsy, particularly with drug-resistant epilepsy.^73^ It also plays a major role in the inflammatory response to infection and cancer. It promotes inflammation and is involved in various autoimmune disorders. IL-12B, also known as IL-12p40, is a common subunit of both IL-12 and IL-23. Interestingly, peripheral blood mononuclear cells (PBMCs) from healthy blood donors^74^ or cord blood from neonates produced both high levels of IL-12 and TNF when stimulated by typical gut commensal gram-positive bacteria, including Bifidobacteria.^75^^,^^76^ Several species and subspecies of Bifidobacteria have been shown to induce TNF production in various *in vitro* models^77^, ^78^, ^79^ of which *B. adolescentis* induced the lowest levels of TNF.^79^ In addition, *B. breve* CNCM I-4035 induced production of various chemokines including CCL3/MIP-1α, TNF and IL-12B in intestinal-like human dendritic cells.^80^ CCL4/MIP-1β was, unfortunately, not measured. In our study, we show that TNF, IL-12B and subspecies of *B. longum* and *B. breve* of patients older than 8 years are significantly higher in responders than non-responders before starting KD and a decrease of these Bifidobacteria positively correlates with a decrease in levels of TNF during KD. Both CCL3 and CCL4 might be involved in the responders-specific signalling between microbiota and inflammation but might not serve as good biomarkers as their expression was not found significantly different between responders and non-responders in our small cohort. Taken together, the results suggest that the cytokine induction by Bifidobacteria is a strain-dependent property.^79^ Bifidobacteria have been found to adhere to human intestinal cells and human mucus *in vitro*^81^^,^^82^ allowing a close interaction with the immune system. Our results indicate an association of these commensal bacteria with inflammation in epilepsy patients and that a decrease of these may alleviate seizures. Thus, patients with higher abundance of members of the *B. longum* cluster and higher levels of TNF may be more likely to benefit from KD treatment. While several studies have shown a direct induction of cytokines *in vitro* by Bifidobacteria, we cannot exclude the possibility that this association may be a result of Bifidobacterial communication via the vagus nerve. It has been demonstrated in mice that *B. longum* NCC3001 could impact the brain by communication via the vagus nerve.^83^

Bifidobacteria are in general regarded as health promoting bacteria important for development of a eubiotic gut^35^ and the development of the immune system in early childhood.^84^ However, a specific *B. longum* – TNF relationship was identified in a large healthy Tanzanian and Dutch cohort where TNF induction was highly dependent on *B. longum* but not *B. adolescentis* in the gut.^85^ Interestingly, *B. longum* abundance was positively associated with urbanization possibly due to dietary habits.

It is intriguing to find early life associated bacteria lingering in the gut of older children with epilepsy. A similar finding in children with autism spectrum disorder (ASD) was recently published.^86^ Wan et al. demonstrated a persistent underdevelopment of the gut microbiota in children with ASD compared to neurotypical age-matched peers. Interestingly, ASD and epilepsy are common comorbidities.

The positive correlation with TNF poses the question whether members of the *B. longum* cluster may be involved in immune dysregulation contributing to the occurrence of seizures in these individuals. Further studies should investigate the role of Bifidobacterial species in larger cohorts including adult patients with epilepsy. Most studies investigating microbial changes in the intestine during KD treatment of epilepsy were performed in children. Comparative whole genome sequencing and *in vitro* characterization of members of both Bifido clusters identified in our study should contribute to our understanding of the functional differences of these subspecies and their role in human health.

Recently, indications that modulation of the gut microbiota could be used to treat epilepsy was found in animal studies as well as limited human studies using either fecal microbila transfers or probiotic treatment.^87^, ^88^, ^89^, ^90^, ^91^ Our study suggests that the reduction of specific Bifidobacteria may reduce seizures in children with refractory epilepsy.

### Strength and weakness of study

While our cohort is larger compared to our pilot study, the sample size is still small. Another issue is the lack of age-matched healthy controls. Nonetheless, we were able to confirm various previous publications, e.g., the increase in relative abundance of *E. coli* and *Bacteroides sp.*, as well as the decrease of Bifidobacteria, IL-17A and TNF during KD. We were able to identify new inflammation-related peptides influenced by KD including those expressed in the gut epithelium and correlating to gut microbes. By combining a panel of inflammatory peptides and measurements of fecal microbiota in our study, we were able to identify known interactions in a new context, i.e., in drug-resistant epilepsy patients, thus, identifying new roles for these interactions. Specifically, we could confirm a correlation between *B. longum/breve* with TNF previously identified by direct interaction in *in vitro* experiments and in a generally healthy cohort.^85^ However, the finding that these Bifidobacteria and TNF were higher in responders before starting KD, especially in older patients, and both decreased during dietary treatment, is novel and may indicate a dysregulation of the gut microbiota in these patients where Bifidobacteria, typically associated with an immature microbiota, are still present and may uphold a certain level of TNF-associated inflammation that could contribute to the higher systemic inflammation triggering the onset of seizures or worsening of seizures. The finding that responders had higher Bifidobacteria and TNF posed the question whether there were differences in baseline nutrient intake before diet. Calculations on consumption of fats, proteins and carbohydrates showed no significant differences between responders and non-responders. Neither was the ratio of fats to proteins and carbohydrates different in relation to response. Thus, differences in macronutrient intake at time of diet start is not likely to explain our findings.

In our study, we only analysed changes in the gut microbiota and inflammation in patient serum. We do not have any data on molecular changes in the brain that might explain the seizure reduction observed. Olson et al. showed that KD treatment of mice reduced seizures through increases in the GABA/glutamate levels in the brain and that a gut microbiota was necessary for this effect.^92^ While TNF may decrease the GABA/glutamate levels in the brain, we can only speculate that this may happen in our cohort. Another possibility is that the overall reduction in systemic inflammation seen in all patients contributes to seizure relief. Downstream consequences may be different for responders and non-responders, e.g., different molecular changes in the brain influenced by the individual cause of epilepsy. However, the identification of the responders-specific *B. longum*/*breve*-TNF signature is intriguing and deserves further study.

To summarize, we here identified a responder-specific *B. longum*/*breve* – TNF association. Treatment with the ketogenic diet reduces both factors, possibly contributing to seizure alleviation. Other inflammatory peptides such as IL-12B, CCL3 and CCL4 might be involved but the mechanisms remain unclear. Our study justifies further investigation to confirm whether either *B. longum/breve*, TNF or both might be useful biomarkers to identify potential responders to KD treatment prior to treatment initiation. In addition, either targeting members of the *B. longum* cluster, TNF or both might reduce seizures without KD treatment. Another intriguing research question that deserves further investigation is why responders to KD, even at older age, retain these early childhood-associated bacteria and whether or how they might contribute to epileptogenesis.

## Contributors

S.P.-N. conceived the idea and designed the experiments. M.D. acquired the ethical permissions and parental consent, was responsible for patient identification, sample collection, diet treatment, and patient data analyses. S.P.-N. and S.S.S. performed the bioinformatics analyses and modelling. A.B. developed the machine learning workflow in MATLAB. S.S.S. and J.D. performed the exploratory ML analysis, A.B. reviewed the code. S.P.-N., S.S.S., M.D., and R.W. wrote the manuscript with support from R.M., A.B., and J.D. Funding was obtained by S.P.-N. and R.M. All authors have read and approved the manuscript.

## Declaration of interests

Authors declare no competing interests.

## Appendix Supplementary materials

**Supplementary Figure 1.** Algorithmic models in the Statistics and Machine Learning Toolbox in MATLAB used in this study. Image, application 1

**Supplementary Figure 2A-C.** ML model evaluations A) Comparing all patients before vs during KD, B) responders vs. non-responders before KD and C) responders vs. non-responders during KD. Image, application 2

Image, application 3

**Supplementary Table 1.** Relative abundance of individual taxonomic profiles Image, application 4

**Supplementary Table 2.** Pre-filtering parameters in HIVE Hexagon to remove host (human) DNA. Only unaligned reads (microbial DNA) were preserved from this step, where these filtered samples were then analysed for their taxonomic composition with CensuScope, and alignments and bacterial relative abundance with HIVE-Hexagon. Image, application 5

**Supplementary Table 3*.*** CensuScope parameters from protocol. Image, application 6

**Supplementary Table 4.** Parameters for the Hexagon alignments from protocol. Image, application 7

**Supplementary Table 5.** MATLAB classification learner parameter selections for Q1, Q3, and Q5. Image, application 8

**Supplementary Table 6-10.** Correlation matrices of multivariate PLS-DA of associations between inflammation markers and gut microbes. These tables underlie the circos plots in Figure 3A–E. Image, application 9

Image, application 10

Image, application 11

Image, application 12

Image, application 13

**Supplementary Table 11.** Proteomics inflammation profile as provided by Olink. Image, application 14

**Supplementary Table 12.** Abbreviations for bacterial species displayed in Figure 3A–E. Image, application 15
