# Supplementary material for: Higher levels of Bifidobacteria and tumor necrosis factor in children with drug-resistant epilepsy are associated with anti-seizure response to the ketogenic diet
Source: eBioMedicine. 2022 May 19;80:104061. doi: 10.1016/j.ebiom.2022.104061 (PMC9126955; doi:10.1016/j.ebiom.2022.104061)

**Supplementary Figure 1**
Algorithmic models in the Statistics and Machine Learning Toolbox in MATLAB used in this study.


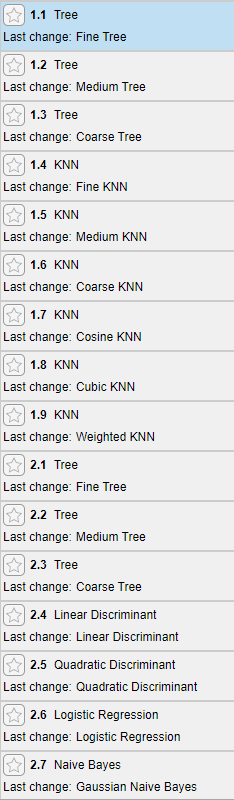

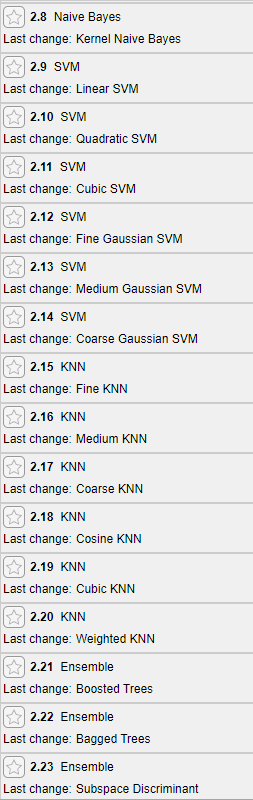

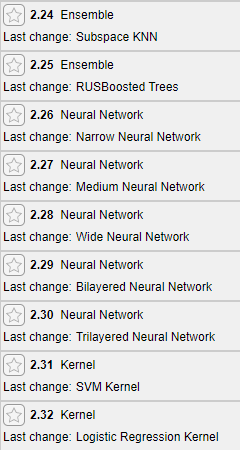

Supplement: Supplementary file 4 — Supplementary Table 1. Relative abundance of individual taxonomic profiles [file mmc4.docx]
