# Supplementary material for: Higher levels of Bifidobacteria and tumor necrosis factor in children with drug-resistant epilepsy are associated with anti-seizure response to the ketogenic diet
Source: eBioMedicine. 2022 May 19;80:104061. doi: 10.1016/j.ebiom.2022.104061 (PMC9126955; doi:10.1016/j.ebiom.2022.104061)
Supplement: Supplementary file 5 — Supplementary Table 2. Pre-filtering parameters in HIVE Hexagon to remove host (human) DNA. Only unaligned reads (microbial DNA) were preserved from this step, where these filtered samples were then analysed for their taxonomic composition with CensuScope, and alignments and bacterial relative abundance with HIVE-Hexagon. [file mmc5.docx]

**Supplementary Figure 2.** ML model evaluations A) Comparing all patients before vs during KD, B) responders vs. non-responders before KD and C) responders vs. non-responders during KD.

| **Supplementary Figure 2A.** Q1 model evaluation metrics (comparing all patients before KD vs. after KD); confusion matrix (2A.1) and AUC/ROC curve (2A.2). The confusion matrix for Q1 assists in evaluating the performance of the classification algorithm by providing a summary of its performance, in this case, the coarse tree algorithm. Each row corresponds to the predicted class, and each column corresponds to the actual  class. By the results shown here, the coarse tree algorithm was able to correctly classify 20 PA (before KD) patients (true positive), falsely classified 3 PA patients (false positive), falsely classified 6 PB (after KD) patients(false negative), and correctly classified 18 PB patients (true negative). The second coarse tree model confusion matrix with percentages reflects the results with expressed ratios. The area under the curve (AUC) and the receiver operating characteristics (ROC) curve are both additional ways to measure the performance of a model. Through the AUC and ROC, the model’s classification performance can be interpreted at various thresholds, where the ROC represents the probability curve and the AUC represents the degree of separability. The y-axis represents the true positive rate (sensitivity) and the x-axis represents the false positive rate (1 - specificity). The closer the AUC is to 1, the better the model, where an AUC of 1 can be interpreted as an excellent model with a good measure of separability. For the coarse tree model chosen for Q1, the AUC is estimated at 0.81, or 81% accurately distinguishing between PA (positive class) and PB (negative class). For the ROC curve, one can interpret values for the ratios of false alarms and hits, where PA can be interpreted as (0.25, 0.87) and PB can be interpreted as (0.13, 0.75), respectively.  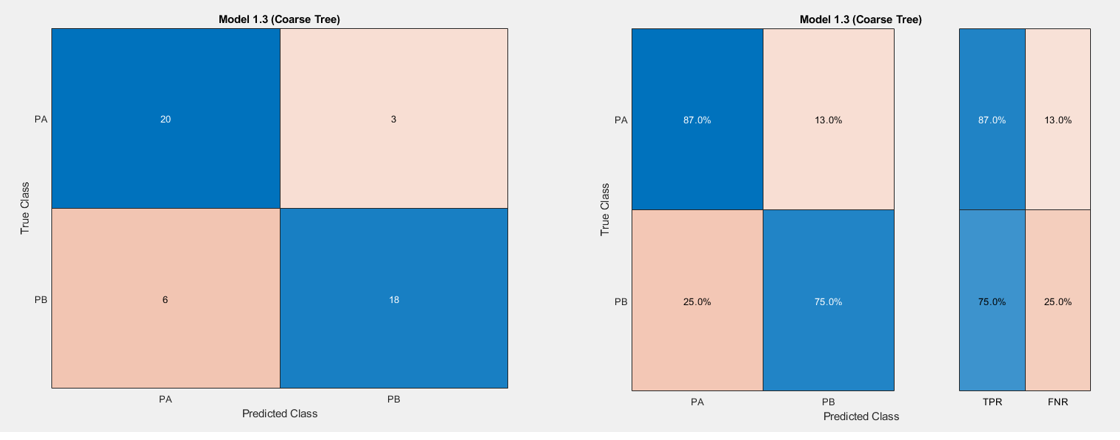  (2A.1)  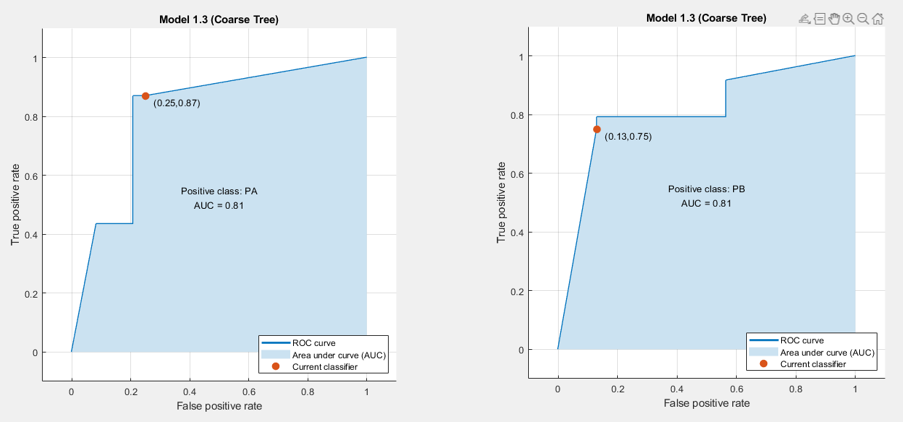  (2A.2)  **Supplementary Figure 2B*.*** Q3 (Responders (R) vs Non-responders (NR) before KD) model evaluation metrics; confusion matrix (2B.1) and AUC/ROC curve (2B.2). The subspace k-nearest neighbors (KNN) algorithm was able to correctly classify 7 Non-Responder patients (true positive), falsely classified 2 Non-Responder patients (false positive), falsely classified 4 Responder patients (false negative), and correctly classified 8 Responder patients (true negative). The second subspace KNN model confusion matrix with percentages reflects the results with expressed ratios. For the subspace KNN model chosen for Q3, the AUC is estimated at 0.71, or 71% accurately distinguishing between NR and R. For the ROC curve, NR can be interpreted as (0.33, 0.78) and R can be interpreted as (0.22, 0.67), respectively.  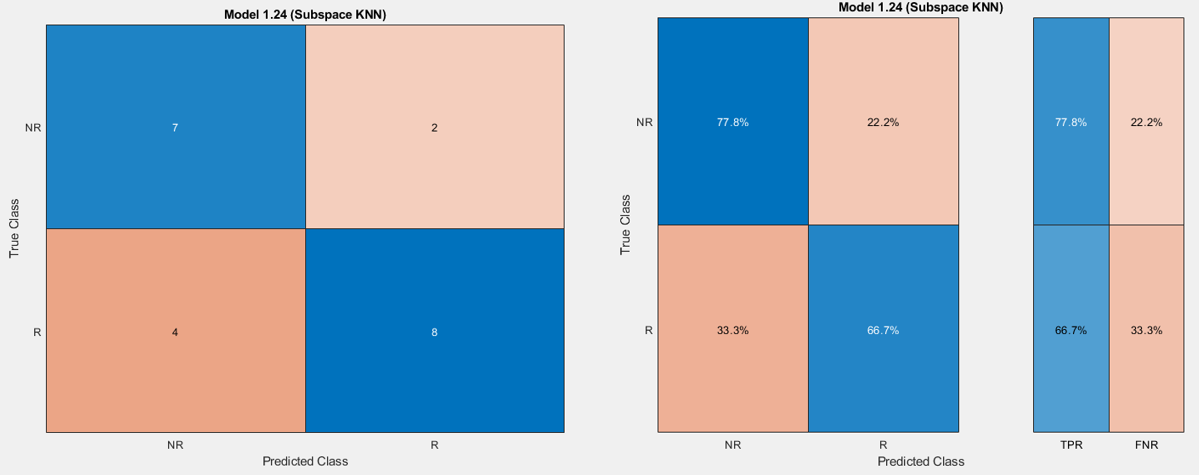  (2B.1)  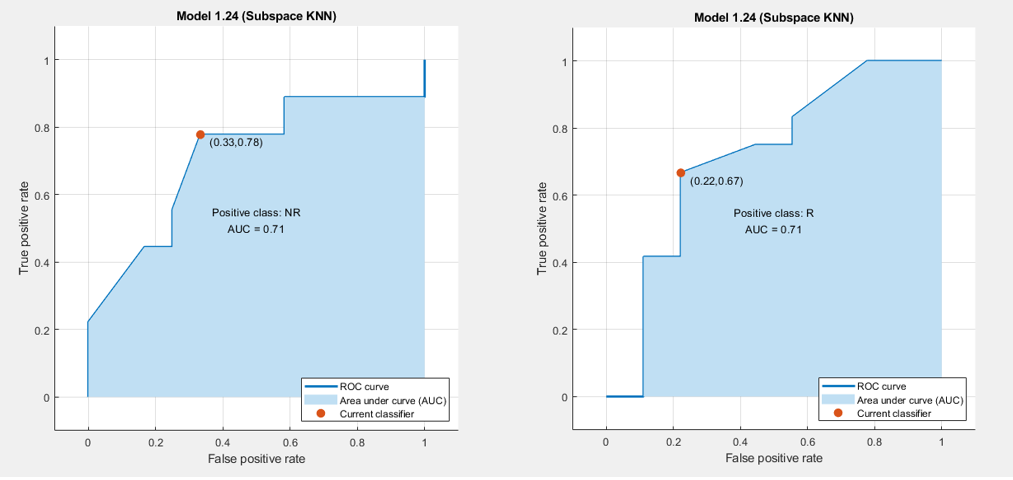  (2B.2)  **Supplementary Figure 2C*.*** Q5 (Responders vs Non-responders during KD) model evaluation metrics; confusion matrix. The Ensemble boosted trees algorithm was unable to correctly classify Non-Responder patients (true positive), falsely classified all 10 Non-Responder patients (false positive), falsely classified Responder patients (false negative), and correctly classified 14 Responder patients (true negative). The second Ensemble boosted trees model confusion matrix with percentages reflects the results as expressed ratios.The AUC/ROC curve could not be generated in MATLAB due to low estimated classification accuracy - essentially this model was unable to correctly classify NR and R patient gut microbiome profiles due to indiscriminate characteristics.  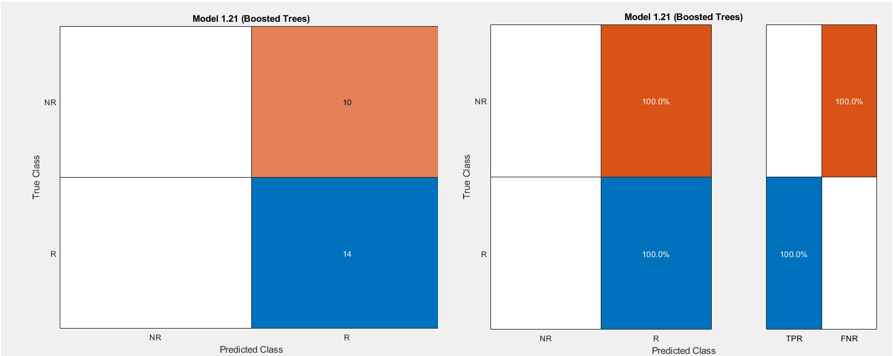 |
| --- |
