# Supplementary material for: Higher levels of Bifidobacteria and tumor necrosis factor in children with drug-resistant epilepsy are associated with anti-seizure response to the ketogenic diet
Source: eBioMedicine. 2022 May 19;80:104061. doi: 10.1016/j.ebiom.2022.104061 (PMC9126955; doi:10.1016/j.ebiom.2022.104061)
Supplement: Supplementary file 6 — Supplementary Table 3. CensuScope parameters from protocol. [file mmc6.docx]

**Supplementary material**

**Supplementary Table 1.** Relative abundance of individual taxonomic profiles

**Supplementary Table 2.** Pre-filtering parameters in HIVE Hexagon to remove host (human) DNA. Only unaligned reads (microbial DNA) were preserved from this step, where these filtered samples were then analysed for their taxonomic composition with CensuScope, and alignments and bacterial relative abundance with HIVE-Hexagon.

**Supplementary Table 3*.*** CensuScope parameters from protocol.

**Supplementary Table 4.** Parameters for the Hexagon alignments from protocol.

**Supplementary Table 5.** MATLAB classification learner parameter selections for Q1, Q3, and Q5.

**Supplementary Table 6-10.** Correlation matrices of multivariate PLS-DA of associations between inflammation markers and gut microbes. These tables underlie the circos plots in Fig. 3A-E.

**Supplementary Table 11.** Proteomics inflammation profile as provided by Olink.

**Supplementary Table 12.** Abbreviations for bacterial species displayed in Fig. 3A-E.

**Supplementary Figure 1.** Algorithmic models in the Statistics and Machine Learning Toolbox in MATLAB used in this study.

**Supplementary Figure 2A-C.** ML model evaluations A) Comparing all patients before vs during KD, B) responders vs. non-responders before KD and C) responders vs. non-responders during KD.
