# Supplementary material for: Higher levels of Bifidobacteria and tumor necrosis factor in children with drug-resistant epilepsy are associated with anti-seizure response to the ketogenic diet
Source: eBioMedicine. 2022 May 19;80:104061. doi: 10.1016/j.ebiom.2022.104061 (PMC9126955; doi:10.1016/j.ebiom.2022.104061)
Supplement: Supplementary file 8 — Supplementary Table 5. MATLAB classification learner parameter selections for Q1, Q3, and Q5. [file mmc8.docx]

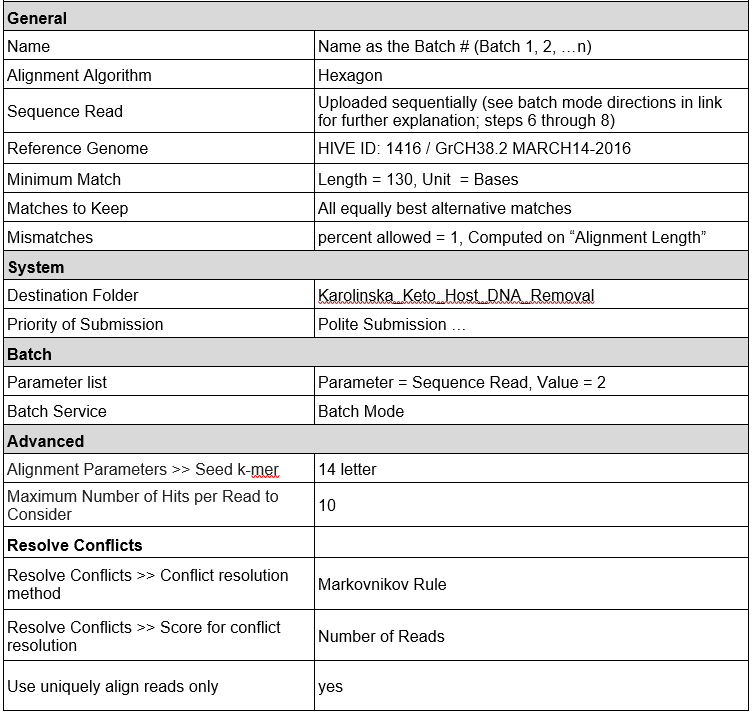


**Supplementary Table 2.** Pre-filtering parameters in HIVE Hexagon to remove host (human) DNA. Only unaligned reads (microbial DNA) were preserved from this step, where these filtered samples were then analyzed for their taxonomic composition with CensuScope, and alignments and bacterial relative abundance with HIVE-Hexagon.
