# Supplementary figures and images for: Higher levels of Bifidobacteria and tumor necrosis factor in children with drug-resistant epilepsy are associated with anti-seizure response to the ketogenic diet
Source: eBioMedicine. 2022 May 19;80:104061. doi: 10.1016/j.ebiom.2022.104061 (PMC9126955; doi:10.1016/j.ebiom.2022.104061)

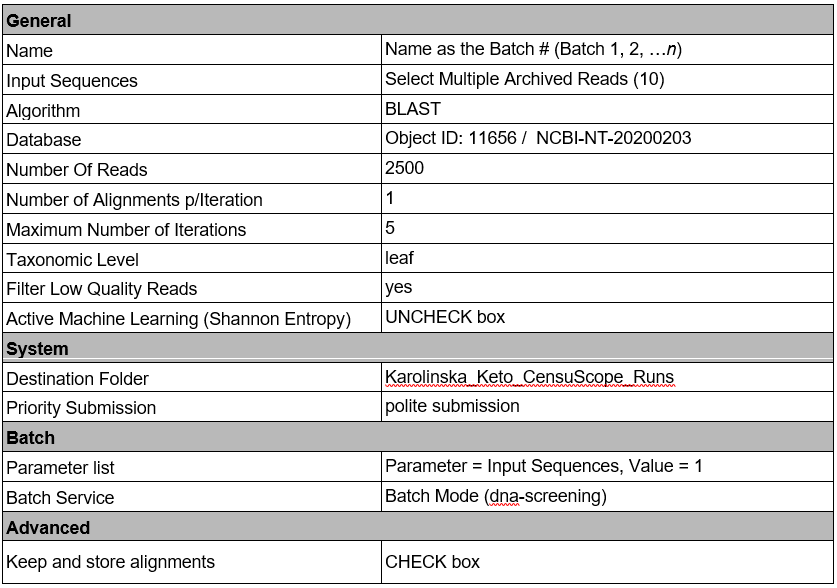


**Supplementary Table 3.** CensuScope parameters from protocol.

Supplement: Supplementary file 9 — Supplementary Table 6-10. Correlation matrices of multivariate PLS-DA of associations between inflammation markers and gut microbes. These tables underlie the circos plots in Figure 3A–E. [file mmc9.docx]

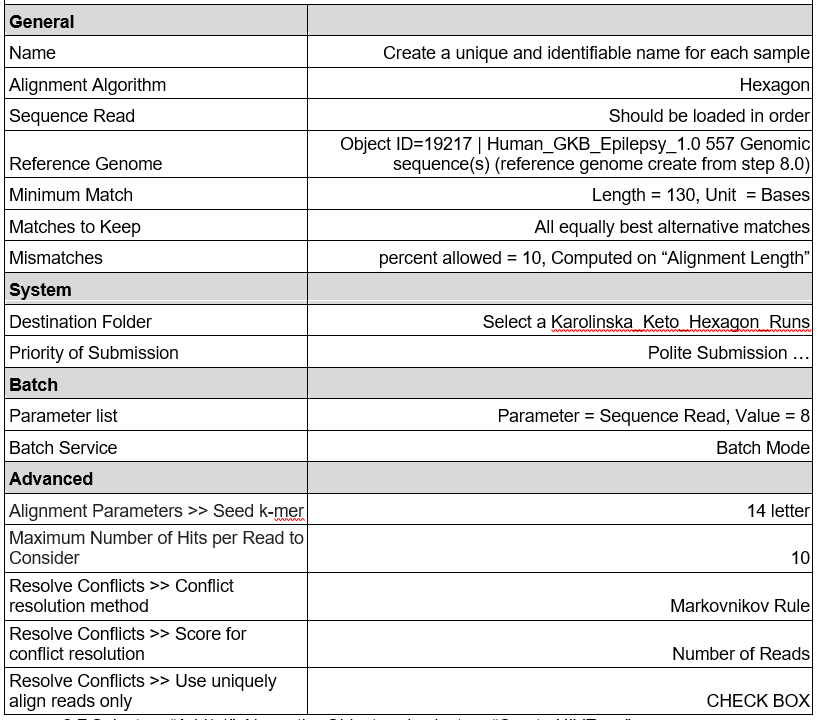


**Supplementary Table 4.** Parameters for the Hexagon alignments from protocol.

Supplement: Supplementary file 10 [file mmc10.docx]
