# Supplementary material for: Higher levels of Bifidobacteria and tumor necrosis factor in children with drug-resistant epilepsy are associated with anti-seizure response to the ketogenic diet
Source: eBioMedicine. 2022 May 19;80:104061. doi: 10.1016/j.ebiom.2022.104061 (PMC9126955; doi:10.1016/j.ebiom.2022.104061)
Supplement: Supplementary file 11 [file mmc11.docx]

| **Question** | **Dataset Variable Array** | **Queried Response** | **# of patients; # of Categories** | **Predictors** | **Validation Method** |
| --- | --- | --- | --- | --- | --- |
| Q1 | 47 x 216 | Microbiome profiles Before KD vs. After KD | 47 Patients; 2 | Bacteria (n = 216) | Cross-validation, *k-folds = 47* |
| Q3 | 21 x 216 | Microbiome Profiles before KD of resulting Responders vs. Non-responders | 21 Patients; 2 | Bacteria (n = 216) | Cross-validation, *k-folds* = 21 |
| Q5 | 24 x 216 | Microbiome Profiles after KD of resulting Responders vs. Non-responders | 24 Patients; 2 | Bacteria (n = 216) | Cross-validation, *k-folds* = 24 |
| **Supplementary Table 5.** MATLAB classification learner parameter selections for Q1, Q3, and Q5. | | | | | |
